# External beam radiation therapy for locally advanced and metastatic gastrointestinal stromal tumors

**DOI:** 10.1186/1748-717X-8-274

**Published:** 2013-11-23

**Authors:** John J Cuaron, Karyn A Goodman, Nancy Lee, Abraham J Wu

**Affiliations:** 1 Department of Radiation Oncology, Memorial Sloan Kettering Cancer Center, 1275 York Avenue, New York, NY 10065, USA

**Keywords:** Gastrointestinal stromal tumor, Radiation therapy, Palliation

## Abstract

**Background:**

The role of radiation therapy (RT) in the management of gastrointestinal stromal tumors (GIST) is not well described. Here we report our institutional experience for patients with locally advanced or metastatic GIST treated with RT.

**Methods:**

Between 1997 and 2012, 15 patients with 22 GISTs were treated with RT at our center. The median age was 68 (range, 41–86). Fourteen patients had stage IV disease and 1 patient had stage IIIB disease, per the American Joint Committee on Cancer (AJCC), 7th Edition staging. Tumors were in a variety of locations, and were most commonly referred for palliative treatment. Eighteen of 22 tumors were symptomatic. Prior to RT, 14 of 15 patients received systemic therapy in the form of tyrosine kinase inhibitors (TKIs) (n = 11), chemotherapy (n = 4), or both (n = 1). TKIs were used concurrently for nine tumors (40.9%). No tumors were treated with concurrent chemotherapy. Several fractionation schemes were used, most commonly 3 Gy × 10 (n = 8). Local progression-free survival and overall survival were estimated using the Kaplan-Meier method. Acute toxicity was graded per Common Terminology Criteria for Adverse Events (CTCAE) v4.0.

**Results:**

The median follow-up was 5.1 months (range, 1.3-28.3). At the time of analysis, 12 patients have died (80%). The estimated 6-month local progression-free survival and overall survival were 57.0% and 57.8%, respectively. Among the 18 symptomatic tumors, at least partial palliation was achieved in 17 (94.4%), and symptoms were completely palliated in eight (44.4%). Treatment was well tolerated, with no Grade 4 or 5 toxicities. There was no Grade ≥3 toxicity associated with concurrent TKI use.

**Conclusions:**

In this largest series to date of GISTs treated with RT, a high rate of palliation was achieved for symptomatic tumors in a cohort of advanced stage, heavily pretreated patients. Treatment was well tolerated, and concurrent use of tyrosine kinase inhibitor therapy was not associated with additional toxicity. While follow-up was short, durable control is possible for some patients, providing evidence that GIST is not universally radioresistant and that RT can provide an important benefit in patients with progressive or metastatic disease.

## Introduction

Gastrointestinal stromal tumors (GISTs) are the most common mesenchymal tumors of the gastrointestinal tract, with an estimated annual incidence of 6.8 per million in the United States [1] and 10 per million worldwide [2]. The management of GISTs consists of surgical resection for localized and potentially resectable tumors, but more than half of patients that undergo complete resection develop recurrence within 5 years [3] and are often treated with systemic salvage therapy. Imatinib, a selective inhibitor of the KIT protein tyrosine kinase, demonstrates superior activity against most GISTs and has led to a dramatic improvement in progression free survival among patients with advanced or recurrent disease [4-8]. Although initial response rates to biologically targeted agents are excellent, many patients develop resistance or metastatic disease, at which point further treatment options are limited.

Data regarding the use of radiation therapy for these tumors is lacking. Several case reports have indicated that radiation can reduce tumor burden and produce durable local control in locally advanced and metastatic tumors [9-20], but, to our knowledge, a robust analysis of its effect in a cohort of patients has not been performed. To further investigate the role of radiation therapy in the treatment of GISTs, we retrospectively analyzed our institutional experience with patients that had locally advanced or metastatic GISTs treated with radiation therapy.

## Materials and methods

### Patient and tumor characteristics

Between 1997 and 2012, a total of 15 patients with 22 GISTs were treated with radiation therapy for either a primary tumor or metastatic disease at our center. These patients were retrospectively identified and placed into a database. Patient characteristics, treatment details, and toxicity information were obtained through chart review. This study was carried out as Study of Existing Data-Application for Exemption from IRB/PB Review, and approval was obtained for a waiver from HIPAA authorization and informed consent as per 45 CFR 164.512(i)(2)(ii) and 45 CFR 46.116(d) (waiver WA0552-12).

The median age of patients was 68 years. The majority of patients had metastatic disease at the time of their radiation treatment, and one patient had stage IIIB disease according to the American Joint Committee on Cancer (AJCC) 7th edition staging. The most common reason that patients were referred for radiation therapy was for palliation of symptoms related to progressive disease (18/22, 82%). Symptoms included pain (n = 12), pain with weakness or numbness (n = 2), bleeding (n = 2), pain with lower-extremity edema (n = 1), and partial bowel obstruction and constipation (n = 1). For those patients that did not have symptomatic tumors, the indications for treatment included the prevention of neurological compromise in the spine (n = 3) and attempted preoperative cytoreduction for an unresectable pelvic tumor (n = 1).

### Radiation therapy

Radiation therapy consisted of megavoltage x-rays delivered by linear accelerator. Tumors were most often treated with 300 cGy × 10 fractions (n = 8). Other conventional fractionation schemes included 180 cGy × 25 and 200 cGy × 25. Stereotactic body radiation therapy (SBRT), defined as hypofractionation of ≥500 cGy per fraction utilizing image guidance for delivery, was used for 9 tumors (2400 cGy × 1, n = 2; 900 cGy × 3, n = 2; 800 cGy × 3, n = 1; 600 cGy × 5, n = 2; and 500 cGy × 5, n = 2).Three patients were treated with a partial course of 300 cGy × 10 but did not complete their course due to clinical deterioration while on treatment. Conventional opposed photon fields were used in the treatment of 13 tumors (59.1%) and IMRT was used for nine tumors (40.9%) in the abdomen, pelvis, and paraspinal region.

### Systemic therapy

Prior to radiation therapy, 14 of the 15 patients received systemic therapy in the form of tyrosine kinase inhibitors (TKIs) (n = 11), chemotherapy (n = 4), or both (n = 1).TKIs that were used prior to radiation therapy included imatinib for all 11 patients, sunitinib in 7 patients, sorafenib in 5 patients, nilotinib in 1 patient and pazopanib in 1 patient. Other systemic agents used before radiation therapy included mesna, doxorubicin, ifosfamide and dacarbazine (MAID) and postoperative mitoxantrone (n = 1), doxorubicin and dacarbazine (n = 1), doxorubicin, paclitaxel and flavopiridol (n = 1), and doxorubicin and vinorelbine (n = 1). Notably, all patients treated with these systemic agents developed progressive disease.

TKIs were used concurrently (imatinib [n = 4], nilotinib [n = 3], sorafenib [n = 1], and sunitinib [n = 1]) with radiation therapy for the treatment of 9 of the 22 tumors, 6 of which (66.7%) were treated with SBRT, No tumors were treated with concurrent chemotherapy.

### Follow-up

Patients were assessed weekly while on treatment. Thereafter, patients were seen at variable intervals by a multidisciplinary disease-management team that generally included surgeons, medical oncologists, and radiation oncologists. The median interval between follow-up visits was 5 weeks (range, 2–12). At each on-treatment visit and follow-up visit, toxicity was assessed per Common Terminology Criteria for Adverse Events (CTCAE) v4.0. Effectiveness of palliation was assessed during on-treatment visits and at the time of follow-up. A patient was considered to have partial palliation if there was any appreciable improvement of symptoms after beginning radiation therapy. Complete palliation was defined as the complete resolution of the presenting symptoms after the beginning of radiation therapy.

Follow-up imaging was available for assessment in 17 of the 22 tumors. Initial radiographic response was assessed according to Response Evaluation Criteria in Solid Tumors (RECIST). The median time to the first radiographic assessment after radiation therapy was 2.2 months.

Local progression was defined as any clinical or radiographic evidence of tumor growth. Overall survival was defined from the date of the first radiation treatment to the date of death from any cause. Local progression-free survival and overall survival were estimated using the Kaplan-Meier method.

## Results

The median follow-up of the entire cohort was 5.1 months (range, 1.4-28.3). At the time of analysis, 12 of the 15 patients (80%) had died. Among the 18 tumors that were symptomatic at presentation and treated with palliative intent, at least partial palliation was achieved in 17 tumors (94.4%). Symptoms were completely palliated in eight tumors (44.4%).

**Figure 1:**
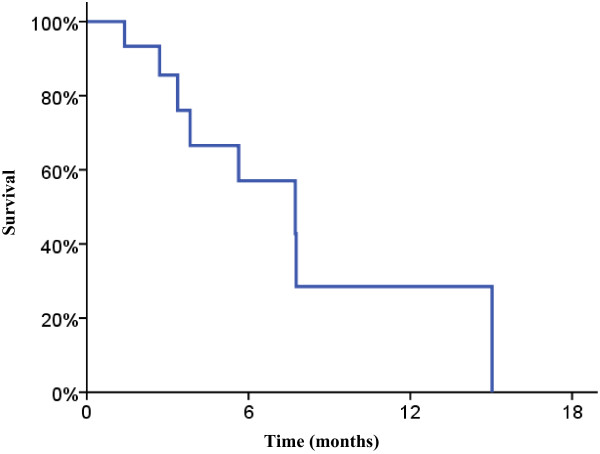
**Local progression free survival.** Kaplan-Meier survival curve for local progression free survival showing estimated 6-month LPFS of 57.0%.

**Figure 2:**
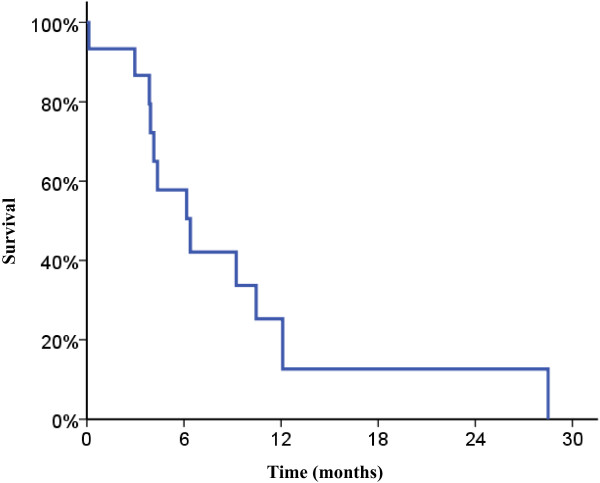
**Overall survival.** Kaplan-Meier survival curve for overall survival showing median survival: 6.6 months and estimated 6 month overall survival of 57.8%.

Partial radiographic response was seen in 35.3% (n = 6) of tumors. Stable disease was seen in 52.9% of tumors (n = 9) and progressive disease in 11.8% (n = 2). Among tumors treated with SBRT with radiographic follow up (n = 8), partial response was seen in 62.5% (n = 5), stable disease was seen in 25.0% (n = 2), and progressive disease in 12.5% (n = 1).The estimated 6 month local progression-free survival was 57.0%, shown in Figure 1. Median survival was 6.6 months, and the estimated 6 month overall survival was 57.8%, shown in Figure 2.

Treatment was extremely well tolerated, with only 1 case of Grade 3 toxicity, consisting of diarrhea in a patient who was being treated to the peritoneum. There were no grade 4 or 5 toxicities. Importantly, there were no Grade ≥3 toxicities seen in patients that were concurrently treated with tyrosine kinase inhibitors. Only one patient experienced persistent dysgeusia after receiving 800 cGy × 3 fractions to the cervical spine. All other toxicities eventually resolved.

A summary of the patient cohort, tumor characteristics, treatment indications and characteristics, outcomes, and toxicities is listed in Table 1.

**Table 1:** Patient, tumor, and treatment characteristics

**Age**	**Sex**	**Tumor location**	**Tumor size (SPD)**	**Indication for RT**	**Symptoms**	**Dose of RT (cGy x Fx)**	Concurrent TKI* therapy	RECIST*	**Degree of palliation**	**Toxicity**	**Grade**	**Status at last follow-up**
56	F	Left flank	50.95	Progression on imatinib	Left flank pain	300 × 10	Y	SD	Partial	Esophagitis	2	A
		Left posterior thorax	12.22	Progression on imatinib	Left back pain	900 × 3	Y	PR	Partial	None	0	
		T12-L1 vertebral bodies	1.2	Progression on imatinib	Left back pain	500 × 5	Y	PR	Partial	None	0	
41	M	L3-L5 vertebral bodies	120	Progression after multiple surgical resections and systemic chemotherapy	Decreased caliber of stools, right hydronephrosis, groin pain, abdominal pain, and left leg edema	180 × 25	N	PD	Partial	None	0	D
67	M	Abdominal wall	45.92	Progression on nilotinib	Abdominal pain	300 × 8	Y	N/A	None	Fatigue	2	D
86	M	Abdomen	94	Progression on Phase I experimental targeted agents	Melena, hematemesis	300 × 10	N	SD	Complete	Nausea, fatigue	1,1	A
73	M	L3-S3 vertebral bodies	N/A	Development of pain while on active surveillance	Left buttock and leg pain	300 × 9	N	N/A	Complete	None	0	D
74	M	T11 vertebral body	4.14	Development of epidural disease while on sunitinib	None	900 × 3	N	PR	N/A	None	0	D
57	F	Liver, anterior abdominal wall	24.91	Progression on nilotinib and sirolimus	Left abdominal and left flank pain	600 × 5	Y	PR	Complete	None	0	D
		C3-C4 vertebral bodies	4.44	Progression on sorafenib	Neck pain, numbness of left shoulder and arm	800 × 3	Y	SD	Partial	Dysgeusia	1	
		C2 vertebral body	N/A	Progression on nilotinib and sirolimus	None	600 × 5	Y	N/A	N/A	None	0	
71	M	Right liver	15.12	Progression on Phase I experimental targeted agents	Shoulder and abdominal discomfort	2400 × 1	N	SD	Partial	Chest wall pain	1	D
69	F	Right ilium and L3 vertebral body	N/A	Progression of symptoms and inability to tolerate systemic therapy	Low back pain, left sciatica, right leg weakness	300 × 10	N	SD	Partial	Diarrhea	2	D
74	F	Peritoneum	142.3	Progression on Sorafenib	Radiographic partial bowel obstruction, nausea, dyspepsia, constipation	300 × 10	N	PR	Partial	Nausea, diarrhea	1,3	D
69	F	Foramen magnum to C2	N/A	Progression on pazopanib	Severe neck pain radiating to occipital scalp and jaw	300 × 10	N	N/A	Complete	None	0	D
		T12 vertebral body to sacrum	N/A	Inability to tolerated Phase I experimental targeted therapy	Right buttock and sacral discomfort	300 × 5	N	N/A	Partial	None	0	
		Left hip	26.95	Progression on pazopanib	Left hip pain	500 × 5	N	PD	Complete	None	0	
68	F	T2-T7 vertebral bodies	N/A	Progression on taxol/flavopiridol	Mid thoracic back pain	300 × 10	N	SD	Complete	Esophagitis	2	D
		C1-C4 vertebral bodies	6.24	Progression on taxol/flavopiridol	None	300 × 10	N	SD	N/A	None	0	
58	M	Left abdominal mass	81.78	Progression on Phase I experimental targeted agents	Hematochezia and hematememsis	300 × 10	N	SD	Complete	None	0	D
45	F	L2-L3 vertebral bodies	8.94	Development of painful symptoms while on imatinib	Lumbar pain exacerbated by sitting	2400 × 1	Y	PR	Complete	None	0	D
64	M	Pelvis	118.3	Attempt for presurgical cytoreduction while on sunitinib	None	200 × 25	Y	SD	N/A	Diarrhea, urinary freq/urgency, fatigue	1, 1, 1	A

*SPD sum product diameter, *cGy × Fx* centigray per fraction, *TKI* tyrosine kinase inhibitor, *RECIST* response evaluation criteria in solid tumors, *PR* partial response, *SD* stable disease, *PD* progressive disease, *A* alive, *D* deceased.

## Discussion

In our retrospective study, the use of radiation therapy achieved a high degree of palliation with minimal toxicity in a cohort of heavily pretreated patients with symptomatic GISTs.

Although a rare tumor, GIST incidence is increasing [2]. The cell of origin is thought to be the intestinal pacemaker cells of Cajal. Tumors can arise in any location along the gastrointestinal tract but are most commonly confined to the stomach and small intestine. The primary therapy for limited resectable disease is surgical resection. However, recurrence is common, and the 5-year disease-free survival is only 45% after surgery alone [3].

Historically, GISTs have been very poorly responsive to traditional cytotoxic chemotherapeutic agents. The discovery that over 90% of GISTs harbor a mutation in one of two tyrosine kinases (KIT and platelet-derived growth factor receptor, alpha polypeptide [PDGFR-a]) led to the widespread use of biologically targeted agents for relapsed or unresectable disease. Imatinib, a selective inhibitor of the KIT protein tyrosine kinase that was developed to treat chronic myelocytic leukemia, was shown to markedly improve relapse-free survival in GIST patients [4-8] and has since emerged as the primary treatment modality for patients that have unresectable or metastatic disease [21]. Results of a recent ACOSOG Phase II trial also support the use of imatinib in the adjuvant setting for high-risk patients [22] by demonstrating improved overall survival compared with historical controls.

Unfortunately, the development of imatinib resistance has become a problem among patients that experience an initial response. Options for the management of resistant disease include dose escalation of imatinib, or switching to other tyrosine kinase inhibitors, which have shown activity in imatinib-resistant disease [23-25]. However, prognosis for these patients remains poor, and progression of disease within the abdomen or at distant sites often causes significant pain and debilitation among a population with a limited lifespan.

For selected patients with focally progressive disease, local therapies such as limited surgical resection [26-29], radiofrequency ablation [30,31], and chemoembolization [32] can provide palliation and durable freedom from progression. Despite clear evidence of benefit from localized therapies, radiation is rarely used in the management of GISTs for either primary or salvage therapy. There appear to be multiple reasons driving this omission. First is the conventional consideration of GISTs as “radioresistant” tumors, perhaps due to their histological relation to soft-tissue sarcomas, which have a relatively slow clinical responsiveness to radiation therapy [33] Secondly, retrospective case series showing a lack of benefit from radiation therapy in the adjuvant setting after surgical resection of GISTs has further reduced enthusiasm for the use of this modality [34,35]. Thirdly, the location of tumors within the abdomen has also limited the ability to deliver high doses of radiation therapy using conventional techniques, due to the radiosensitivity of surrounding organs. Finally, physicians may be hesitant to temporarily discontinue TKI therapy to administer a course of palliative radiation due to concerns of disease progression at other sites. While simultaneous administration is an option, the increased risk of high-grade dermatologic and mucosal toxicity when other TKIs (targeted against the epidermal growth factor receptor) are used with radiation therapy [36] often deters physicians from recommending concurrent treatment.

Recently, new insights into the vast genetic and molecular heterogeneity of sarcomas, coupled with modern organ-sparing radiation techniques, have challenged the assumptions about radiation therapy in the management of this disease. Several case reports throughout the literature have shown significant clinical and radiographic responses with the use of radiation therapy, with the ability to maintain long-term disease control and palliate bone and visceral metastases in certain cases [9-20]. However, these studies are limited by small patient numbers and the inherent selection and publication biases of single patient reports. In the current study, all patients that were treated consecutively at a single institution were included, making for a more robust analysis, and representing the largest report to date. Results from the current study corroborate previous case reports, as radiation therapy was shown to be highly effective in achieving at least partial palliation in the majority of symptomatic patients. An initial radiographic response was seen in over a third of tumors with adequate follow-up imaging. Toxicities were largely related to the site being treated, were generally mild, and the majority resolved shortly after treatment. In regard to TKI-related toxicity, there were nine tumors that were treated with concurrent tyrosine kinase inhibitor therapy. Among these patients, there were no Grade ≥3 toxicities and no dermatologic toxicities, consistent with previous phase I and II trials demonstrating the safety of concurrent TKI therapy (albeit at a reduced daily dose) and radiation therapy for oligometastases [37,38] The results of the current study suggest that, for those patients that are benefitting from TKI therapy at some sites of disease but progressing at others, concurrent radiation therapy can be safe and effective in helping to control symptoms of pain, weakness, and obstruction. Collectively, these results indicate that radiation therapy can provide both objective responses and symptomatic relief without detracting dramatically from quality of life, and should be considered in the multidisciplinary care of patients with locally advanced or metastatic GISTs.

Our study is limited by its retrospective design. Because the follow-up schedule of the cohort was heterogeneous, conclusions regarding radiographic response and toxicity are limited. Further, the non-uniformity of the radiation doses makes it difficult to analyze dose response and effectiveness of certain dose levels. Finally, the follow-up for the cohort is short, reflecting the poor prognosis of these advanced-stage, heavily pretreated patients. Despite these limitations, the findings remain hypothesis generating and give important insights into a treatment modality that appears to be safe and effective in the management of patients with metastatic or locally advanced GISTs.

## Conclusions

This study represents the largest report of patients with locally advanced and metastatic GISTs treated with radiation therapy. Radiation was largely effective at achieving palliation for symptomatic tumors, and radiographic response is possible in some patients, providing evidence that GIST is not universally radioresistant. Toxicities were mild, and concurrent use of TKI therapy did not portend an increased risk of side effects. Further study to establish the role of radiation therapy in the management of gastrointestinal stromal tumors is needed and supported by the findings of this report.

### Consent

This study was carried out as Study of Existing Data-Application for Exemption from IRB/PB Review, and approval was obtained for a waiver from HIPAA authorization and informed consent as per 45 CFR 164.512(i)(2)(ii) and 45 CFR 46.116(d) (waiver number WA0552-12).

## Competing interests

The authors declare that they have no competing interests.

## Authors’ contributions

JJC participated in the design of the study, carried out the chart review, performed statistical analyses, and drafted the manuscript. KAG and AJW participated in the design of the study and helped to draft the manuscript. NL conceived of the study and participated in the revision of the manuscript. All authors read and approved the final manuscript.
